# Serum exosomal pregnancy zone protein as a promising biomarker in inflammatory bowel disease

**DOI:** 10.1186/s11658-021-00280-x

**Published:** 2021-08-10

**Authors:** Jing Shao, Yan Jin, Chunhong Shao, Hui Fan, Xiaorui Wang, Guang Yang

**Affiliations:** 1 Department of Clinical Laboratory, Shandong Provincial Hospital Affiliated to Shandong First Medical University, Jinan, 250021 Shandong China; 2 Department of General Surgery, The 960th Hospital of the PLA Joint Logistics Support Force, Jinan, 250031 Shandong China

**Keywords:** Inflammatory bowel disease, Exosome, Proteomics, Pregnancy zone protein

## Abstract

**Background:**

Inflammatory bowel disease (IBD) is a kind of intestinal immune dysfunction disease, and its occurrence and prevalence are on the rise worldwide. As a chronic gastrointestinal disease, its pathogenesis is still unknown. Exosomes are vesicles in various body fluids that carry a variety of substances. They can mediate intercellular communication and long-distance transport of multiple media. In this study, we investigated the protein profile of serum exosomes from healthy people and IBD patients to explore a new serological biomarker for IBD.

**Methods:**

Initially, exosomes were extracted from serum samples, and the proteins within the exosomes were identified by label-free liquid chromatography/mass spectrometry (LC-MS/MS). Western blot and ELISA were used to assess the identified protein. To further analyze the target protein, an acute colitis mouse model was established, and exosomes in colonic tissue and serum were extracted to investigate the protein in them.

**Results:**

Firstly, serum exosomes were extracted from samples, and proteins in exosomes were identified by LC-MS/MS. Through statistical analysis, we identified 633 proteins. Among these proteins, pregnancy zone protein (PZP) showed a marked difference between patients with IBD and healthy people, in that its expression level was much higher in the IBD patients This exosomal protein was associated with immunosuppressive effects. Also, the level of PZP in colon tissue exosomes and serum exosomes of acute colitis mice was significantly higher than that of the control group.

**Conclusions:**

Our findings indicated that serum exosome PZP was present at a high level in the IBD patients. Hence it might be a promising biomarker and enhance auxiliary diagnosis of IBD.

## Background

Inflammatory bowel disease (IBD) is a prototypical complex disease. It involves two disabling immune-mediated conditions: Crohn’s disease and ulcerative colitis [1]. It has high prevalence worldwide and is particularly common in Asia [2]. The annual recurrence rate of IBD patients in China is about 0.0145–0.0196%. Young and middle-aged patients may have serious complications, bringing a heavy economic burden to their families and society [3]. Previous reports have shown that numerous factors, such as environment, genes, diet, and microbiota, interact in an intricate way, leading to the biological complexity of IBD [4]. Therefore, the pathogenesis of IBD still needs to be studied.

At present, the diagnosis of IBD is based on a combination of clinical, endoscopic, biochemical, cross-sectional imaging, and histological examination [5], most of which are invasive detection methods. However, in general, the diagnosis of IBD is still significantly delayed, resulting in a delayed start to treatment, which harms the patient’s health and disease progression [6]. Furthermore, a single reference criterion for the diagnosis of IBD does not exist [7]. Given the current high primary and acquired resistance to IBD treatment, the emergence of new drug targets and biomarkers is urgently expected.

Exosomes are small single-membrane vesicles (30–150 nm size) derived from most cells into the extracellular space, enriched for various biological components, such as lipids, nucleic acids, proteins, and glycoconjugates [8, 9]. Many kinds of cells can secrete exosomes. They act locally or have comprehensive effects through their circulation in various physiological fluids, such as serum, saliva, urine, etc. [10]. Reports on exosomes have increased. The data showed that exosomes could be regarded as modulators in many diseases, such as diabetes, cardiovascular disease, coagulation disease, polycystic ovary syndrome, and autoimmune diseases [11, 12]. In recent years, the role of exosomes in the pathogenesis and treatment of IBD has become a hotspot. For example, Wong et al. isolated and studied serum exosomes from mice with acute colitis [10]. Serum exosomes could significantly activate macrophages in vitro, which suggested the potential of serum exosomes in IBD diagnosis. In addition, Zheng et al. extracted exosomes from the saliva of patients with IBD. The analysis showed that the expression of PSMA7 in IBD patients was significantly higher than that in healthy controls [13]. Because of the extensive existence of exosomes in these biological fluids and the various substances in them, especially the cell-type-specific proteins and genetic material, they could be used as biomarkers and indicators in diagnosing disease [14].

Proteomics is an effective means to identify biomarkers and provides a new opportunity to search for novel, compassionate, and specific markers in body fluids to detect IBD. In this study, we collected serums from healthy people and IBD patients and extracted serum exosomes. In addition, LC-MS/MS was performed for serum exosome protein profiling, contributing to finding new indicators and new therapeutic targets for IBD.

## Methods

### Study population and sample collection

From January 2019 to June 2021, 45 patients diagnosed with IBD were recruited in Shandong Provincial Hospital Affiliated to Shandong First Medical University. Also, 45 healthy subjects without IBD were recruited as controls. These 45 patients were newly diagnosed with IBD and had no treatment. They were matched with the control group in terms of age, gender, and systemic diseases. Subjects with the diagnosis of diabetes, autoimmune diseases, and other common diseases, or any related inflammatory or infectious diseases (such as tuberculosis or cytomegalovirus, urinary tract infection, etc.) were excluded from the two groups. Blood samples were taken from each participant and collected in tubes without anticoagulants. Blood samples were centrifuged at room temperature at 3000 rpm for 15 min. Serum was collected, sub-packed, and stored in a refrigerator at **–**80℃ until use.

### Serum exosome isolation

As described in previous reports, serum exosomes were isolated by multi-step centrifugation [15, 16]. The collected serum was centrifuged at 300*g* for 10 min at 4 ℃ to remove floating cells, and then centrifuged at 820*g* for 15 min, 10,000*g* for 5 min at 4 ℃ , and passed through a 0.8-µm syringe filter to remove cell debris. The extracellular exosomes (size, < 1 μm) were pelleted in a final centrifugation at 100,000*g* for 2 h at 4 ℃. Pelleted exosomes were resuspended in PBS at a ratio of 25 µL of PBS per 100 µL of serum. The BCA protein assay kit determined the protein concentration in exosomes (Thermo Scientific, Product #23,225).

### Transmission electron microscopy

Exosome extraction was performed according to the protocol of the Invitrogen Total Exosome Isolation kit. We used electron microscopy to identify the shape and size of the substances extracted and confirm exosomes’ isolation. The extracted exosomes were resuspended in 1× PBS. Then, aliquots (5 µL) of the exosome samples were placed on carbon-coated grids (previously treated with plasma cleaner; Ted Pella Inc, CA, USA). The samples were blotted with filter paper after 30 s. Then the samples were stained with 2% uranyl acetate for 1 min. The grids were examined under the FEI T12 electron microscope at 120 kV. The micrographs were taken using a Gatan Ultra scan of 4K×4K.

### Nanoparticle tracking analysis (Nanosight)

The size of serum exosomes was analyzed using the Nanosight LM10-HS system (Nanosight Ltd., Amesbury, UK). Briefly, exosome pellets resuspended in PBS were diluted to a concentration of 3 µg/µL after protein quantification. The exosome suspensions were further diluted 100-fold and analyzed following the manufacturer’s protocol.

### LC-MS/MS analysis and protein identification

Five samples were selected from each group for mass spectrometry analysis. Each sample was separated by a nanoliter flow rate Easy nLC system. Buffer A was 0.1% formic acid aqueous solution, and buffer B was 0.1% formic acid acetonitrile aqueous solution (acetonitrile 80%). The chromatographic column was equilibrated with 100% buffer A, and the sample was loaded onto the analytical column (Thermo Fisher Scientific, Acculam PepMap RSLC 50 μm × 15 cm, nano viper, P/ N 164,943) by the automatic injector for separating. The flow rate was 300 nL/min. The 2-h liquid phase gradient: 0**–**5 min, B solution 3%; 5**–**95 min, the linear gradient of B solution was from 3 to 28%; 95**–**110 min, the linear gradient of liquid B was from 28 to 38%; 110–115 min, the linear gradient of liquid B was from 38 to 100%; 115**–**120 min, B solution maintained at 100%.

The samples were separated by chromatography and analyzed by a Q Exactive Plus Mass Spectrometer. The resolution of the first mass spectrum is 70,000 and that of the second mass spectrum is 17,500. The full scan was carried out in the orbit of 350–1800 m/z. After each full scan, the top 10 most intense ions were automatically selected as high energy collision dissociation (HCD) fragments, the isolation window was 2 m/z, and the normalized collision energy was 27%. Typical mass spectrum conditions were as follows: automatic gain control (AGC) target was 3 × e6 ions for full scan and 1 × e5 MS/MS scanning; the maximum injection time of the first stage was 50 ms, and the maximum injection time of the second stage was 45 ms.

Proteins were identified and quantified against the complete human proteins in the UniProt database (Uniprot_HomoSapiens_20367_20200226) using MaxQuant software (version 1.5.5.1) for database search and the Label-Free Quantification (LFQ) algorithm for quantitative analysis [17]. The parameters were set as follows: a maximum of two missed cleavages was allowed; the main search was set as 4.5 ppm; the first search was set as 20 ppm; MS/MS tolerance was set as 20 ppm; carbamidomethylation of cysteines was considered as a fixed modification, and the oxidation of methionine and protein N-terminal acetylation were classified as variable modifications; peptide false discovery rate (FDR) value ≤ 0.01; protein FDR ≤ 0.01.

### Western blot analysis

Western blot and relative protein quantity analysis were performed as described previously [18]. Briefly, protein extracts were applied to SDS-polyacrylamide gel and transferred to a nitrocellulose membrane. After incubating with primary antibodies and appropriate secondary antibodies, the immunoreactive bands were chromogenously developed with 3,3′-diaminobenzidine. The relative quantity of proteins was analyzed by Quantity One software (Bio-Rad, Hercules, CA, USA) and normalized to GAPDH or β-actin levels. The primary antibodies anti-CD63 (#25682-1-AP), TSG101 (#14497-1-AP), HSP70 (#66183-1-Ig), GAPDH (#60004-1-Ig), and β-actin (#20536-1-AP) were all from Proteintech Group, Inc, and the anti-PZP (#PA5-110249) was from Invitrogen, USA. The secondary antibodies Anti-Rabbit IgG (#S0001) and Anti-Mouse IgG (#S0002) were both from Affinity Biosciences, OH, USA.

### Animal studies

Male C57BL/6 mice (6–8 weeks old, weighing 18–22 g) were from the Animal Research Center of Peking University People’s Hospital and were housed in a specific pathogen-free facility. All surgical interventions and postoperative animal care were performed with the permission of Shandong Provincial Hospital Affiliated to Shandong First Medical University Ethics Committee. All mice were group housed with an air-conditioned temperature of 20 ± 2 ℃, a humidity of 55 ± 10%, and a light/dark cycle of 12/12 h. The mice were treated with 4% dextran sulfate sodium (DSS) in drinking water for seven consecutive days to induce colon injury and colitis [19]. Normal control mice were given normal drinking water. Mice were sacrificed on day eight by eye bloodletting followed by cervical dislocation. The colons were mechanically separated and cleaned, and the length was measured. The mouse colon tissues were subjected to lysis by RIPA for further study.

### Body weight measurement and blood and tissue collection

Body weight of mice was measured during treatment, and plasma lipid profiles were measured at the end of the experiment. Food was removed for an eighth fast, then blood was collected from the inferior vena cava, and animals were killed by exsanguination. The heart and aorta were rapidly removed after perfusion with ice-cold PBS. The aortic root and brachiocephalic artery of mice were snap-frozen in optimal cutting temperature embedding medium for histology and immunofluorescence assay. The remaining aorta was opened longitudinally and fixed with 10% buffered formalin for measuring the surface area covered by lipid-staining lesions. Brachiocephalic arteries were removed for further analysis.

### H&E staining of colonic tissue

Mouse colons were fixed at room temperature in 10% formalin for 24 h and then embedded in paraffin. According to the standard protocol, the tissue sections were 5 μm thick and stained with hematoxylin and eosin (H&E). Images were collected using a Nikon Eclipse Ci-L microscope and viewed by 3DHISTECH CaseViewer2.2 (Hungary).

### ELISA

100 uL of serum-derived exosomes were resuspended in 100 uL of ELISA lysis buffer (100 mM Tris-HCl, pH7.4, 150 mM NaCl, 1 mM EGTA, 1 mM EDTA, 1% Triton X-100, 0.5% sodium deoxycholate, with protease inhibitor cocktail added immediately before use) [20]. PZP was measured using a commercial ELISA kit (R&D Systems, Minneapolis, MN, USA). Validation was performed according to published recommendations [21].

### Data analysis

Proteins were mapped to their corresponding cellular locations, biological processes, and molecular functions by DAVID (Database for Annotation, Visualization, and Integrated Discovery) software v6.7 (http://david.abcc.ncifcrf.gov/). ImageJ software was used to assess differences in Western blotting results among groups. Data were analyzed by one-way ANOVA (followed by the Scheffé F test for post hoc analysis). A value of *p* < 0.05 was considered statistically significant.

## Results

### Exosome extraction and identification

**Fig. 1:**
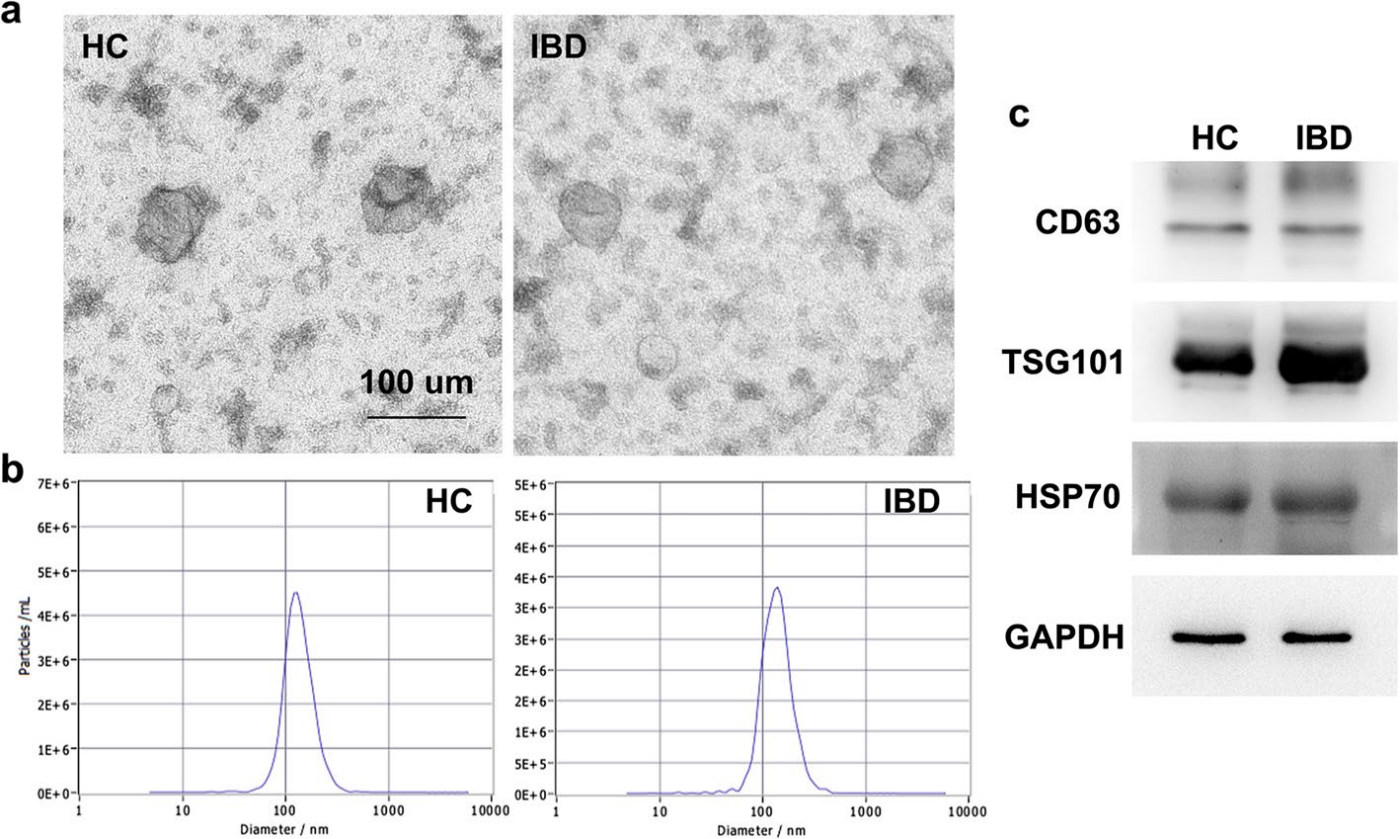
Characterization of exosomes. **a** Electron microscopy to identify the characteristics of serum exosomes derived from HC and IBD patients; **b** NAT of exosomes; **c** CD63, TSG101, and HSP70 expression of exosomes was detected using Western blot assay

An exosome isolation kit was used to extract exosomes from serum samples, and electron microscopy was used to identify the shape and size of the substances extracted and confirm exosome isolation. The electron microscopy images of serum exosomes from healthy controls (HC) and IBD patients (referred to simply as IBD) are shown in Fig. 1a. The exosomes were spherical, primarily vesicles with a diameter of 30–120 nm. The bilayer lipid membrane could be easily observed. Next, Nanoparticle Tracking Analysis (NAT) was performed to measure the diameter distribution of these exosomes. As shown in Fig. 1b, the diameter of isolated exosomes had a single peak at approximately 100 nm (the exosome peak in HC and IBD was 127.8 nm and 135.6 nm, respectively). The purified exosome-specific markers, including CD63, TSG101, HSP70, were detected by Western blot (Fig. 1c). It showed that these markers were commonly and highly expressed in purified serum exosomes. The results illustrated that we have successfully isolated and identified exosomes from serum samples.

### Serum exosomal proteins in HC group and patients with IBD

**Fig. 2:**
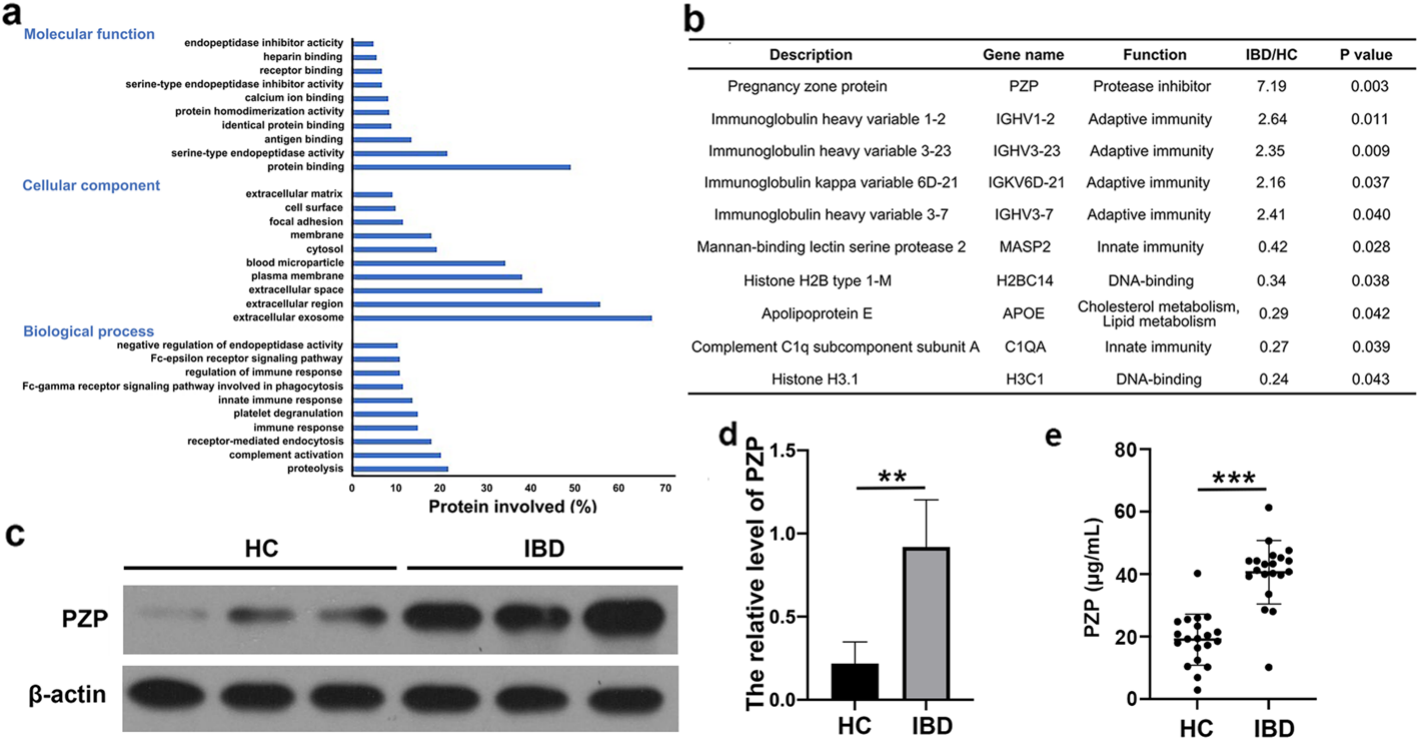
Proteins in serum exosomes. **a** GO analysis of the total 633 differentially expressed proteins was based on the LC-MS/MS results in the aspects of molecular function, cellular component, and biological process; **b** 10 proteins were significantly differentially expressed; **c** Western blot showed that the level of PZP in serum exosomes of IBD patients was significantly increased, n = 10; **d** Relative level of PZP. **p < 0.01; **e** ELISA showed that the level of PZP in serum exosomes of IBD group was higher than HC group, unpaired t-test, n = 30, ***p < 0.001

LC-MS/MS was performed for serum exosome protein profiling. We identified a total of 633 proteins and 292 proteins expressed differentially in the HC and IBD groups. DAVID functional annotation was utilized to analyze the 292 differentially expressed proteins in serum exosomes. As a result, 285 proteins were identified from 292 proteins and classified into corresponding molecular functions, cell components, and biological processes, according to the GO (Gene Ontology) database (Fig. 2a). GO molecular function analysis indicated that proteins were involved in protein binding (49%), serine-type endopeptidase activity (21%), and antigen binding (13%). GO cell component analysis demonstrated that serum exosome proteins were located in the extracellular region (55%), cytosol (19%), immunoglobulin complex (3.5%), and lipoprotein particles (8%). GO biological process analysis suggested that the proteins were involved in the response to proteolysis (21%), complement activation (20%), receptor-mediated endocytosis (18%), the acute immune response (15%), and platelet degranulation (15%). The results are similar to those reported in previous articles [10, 22].

In the significant difference analysis of quantitative results, we screened the data with at least two non-null values in the three repeated experimental data in the sample group for statistical analysis. Among them, proteins that expressed a differential multiple greater than 2.0 (up or down) and a P-value (t-test) less than 0.05 were considered differentially expressed proteins. After screening, ten proteins met the above criteria, five proteins were up-regulated, and five were down-regulated (Fig. 2b). Among the ten proteins, PZP was distributed in most GO terms with a high enrichment fraction. PZP is a broad-spectrum immunosuppressant with anti-protease activity. It was thought to have a role in pregnancy by inhibiting the cell-mediated immune response to prevent fetal rejection [23]. In addition, PZP has been shown to inhibit the immune reactivity of T lymphocytes, recruitment, migration, proliferation of T cells, and IL-2 production [24, 25]. However, the role of PZP in IBD has rarely been reported. Therefore, we took PZP as the target protein and conducted studies in the IBD model.

### Verification of PZP expression in serum exosomes

Western blot and ELISA were used to verify the PZP levels in serum exosomes of HC and IBD groups. Compared with healthy people, the serum exosomes of IBD patients were significantly increased (Fig. 2c**–**e). The results were consistent with LC-MS/MS.

### DSS-induced acute colitis mice model

**Fig. 3:**
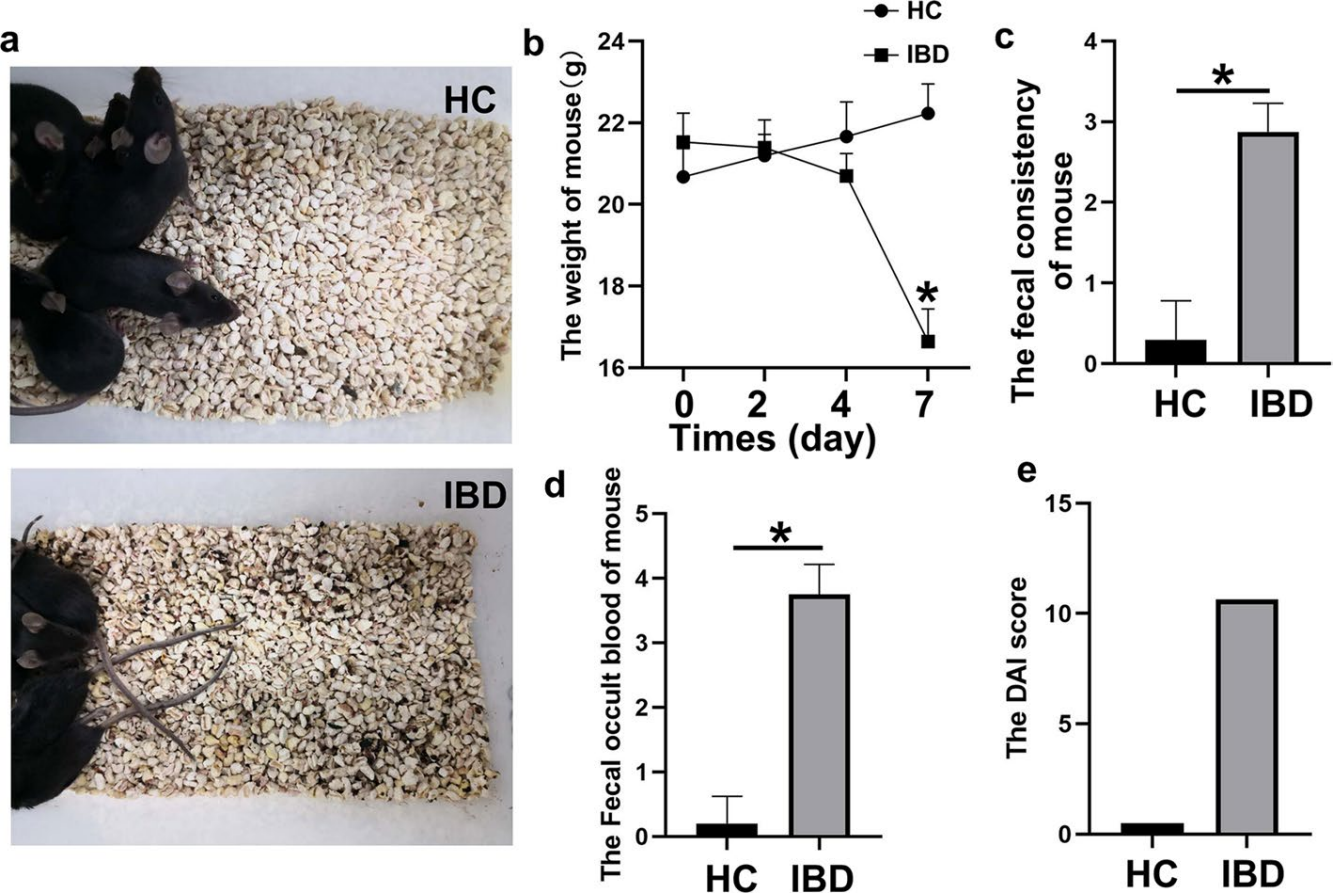
DSS-induced acute colitis mouse model. **a**–**d** Body weight, fecal consistency, and fecal occult blood of mice were measured; **e** DAI score in the DSS group (10.625) was significantly higher than that in the control group (0.500), n = 10, *p < 0.05

The dextran sulfate sodium (DSS) induced colitis mouse model is widely used in the study of IBD. The DSS model is superior to other animal models because of its high reproducibility and similarity to disease manifestations in patients with IBD [26]. The model of HC and IBD was established with the male C57BL/6 mice. To evaluate the effect of 4% DSS administration, body weight, fecal consistency, and fecal occult blood of mice were measured. The diet and water intake, mental state, activity, and fecal characteristics of mice in the control group were normal, and body weight was increased. Mice in the DSS group began to show reduced activity, reduced dietary water intake, wet and cold body hair, loose stools, gross blood stools, and moist and oozing blood around the anus on day 4. The body weight of the DSS group [(16.64 ± 0.80) g] was significantly lower than that of the control group [(22.23 ± 0.72) g]. The average body weight was decreased by 25% on day 8 compared with their initial body weight at the start of the experiment, and two mice in the DSS group died. The DAI score in the DSS group (10.625) was significantly higher than that in the control group (0.500) (Fig. 3).

**Fig. 4:**
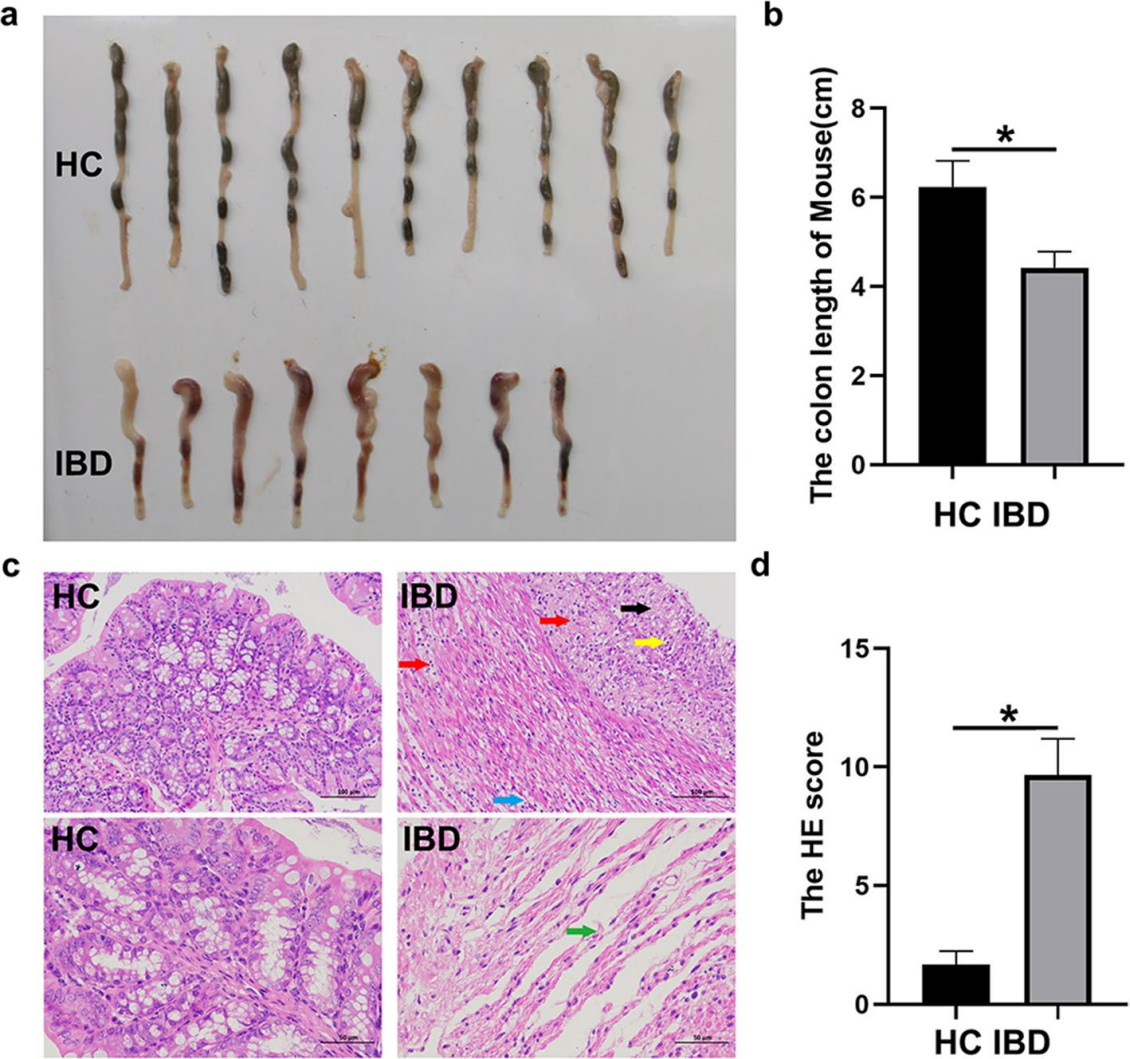
4% DSS consumption for 7 consecutive days can successfully induce acute colitis in mice. **a–b** Colon length of HC and IBD groups; **c–d** The H&E score was increased from 1.67 ± 0.58 in the control group to 9.67 ± 1.53 in the DSS group, n = 10, *p < 0.05

The mice were sacrificed on day 8. Then colon sections were removed, and their phenotype was measured. The average colon length of the DSS group was significantly reduced by 29%, from 6.24 ± 0.58 cm in the control group to 4.42 ± 0.36 cm (Fig. 4a, b). Histological features of the colon evaluated by H&E staining revealed that the DSS-induced colitis mouse model showed signs of significant inflammation. In the control group, the colonic mucosa was intact, the glands were arranged neatly, the crypt structure was standard, and there was no reduction of goblet cells. Only a few inflammatory cells were infiltrated in some mice. Colonic mucosal defects, incomplete glands, crypt shortening or disappearance, reduced goblet cells and inflammatory cell infiltration were significantly increased in the DSS group. The H&E score was increased from 1.67 ± 0.58 in the control group to 9.67 ± 1.53 in the DSS group (Fig. 4c, d). The results suggested that 4% DSS consumption for seven consecutive days can successfully induce acute colitis in mice.

### PZP expression in DSS-induced mice

**Fig. 5:**
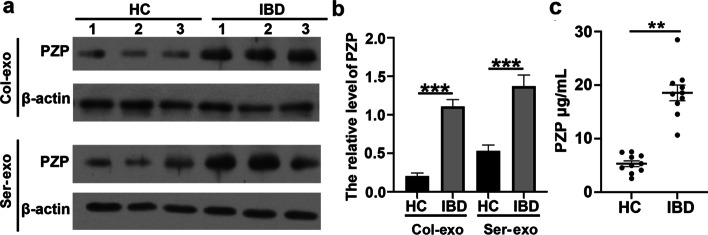
IBD levels were elevated both in the colon tissues exosomes (Col-exo) and serum exosomes (Ser-exo) of acute colitis mice. **a** Compared with the control group, PZP expression was higher in colon tissue of the IBD group; **b** Relative level of PZP, n = 10, ***p < 0.001; **c** ELISA showed that the level of PZP in serum exosomes of the IBD group was higher than the HC group, unpaired t-test, n = 10, **p < 0.01

Western blot was used to detect the expression of PZP protein in exosomes of colon tissues and serum (Fig. 5a, b). Compared with the control group, PZP expression was higher in the exosomes of colon tissues and serum in the IBD group. Moreover, the expression of PZP in the serum exosomes of mice was also detected by ELISA. Compared with the control group, the level of PZP in the serum exosomes of the IBD group was higher (Fig. 5c). These results suggested that the elevation of PZP in serum exosomes was consistent with the level of PZP in colonic tissue exosomes, which further illustrated that the level of PZP could be used as an indicator of IBD.

## Discussion

This study found that PZP, previously described as a serum protein elevated in the blood of pregnant women, was expressed in serum exosomes and was significantly elevated in patients with IBD. It could be used as a potential biomarker for the diagnosis of IBD.

There is a lack of useful serological markers in the screening and auxiliary diagnosis of IBD. Serum is easy to obtain as an auxiliary diagnostic specimen. Serum protein markers, as potential biomarkers, are of great significance. To be a diagnostic and prognostic biomarker is one potential clinical application of exosomes. Exosomes exist in various body fluids and may reflect the state of their parental cells. Thus, exosomes are ideal non-invasive diagnostic markers [27]. Reports showed that exosomes expressing CD63 and caveolin-1 in plasma could be considered non-invasive markers of melanoma and a new tool for clinical treatment of cancer patients [28]. Exosomes are associated with a variety of inflammatory diseases, such as juvenile idiopathic arthritis [29], sarcoidosis [30], and asthma [31]. Therefore, finding differentially expressed proteins in circulating exosomes of IBD patients is helpful for the diagnosis and treatment of IBD.

Mass spectrometry is one of the most effective quantitative analysis platforms with the advantages of robustness, sensitivity, selectivity, multiplexing, and high throughput [32]. Quantitative proteomics based on LC-MS/MS has become an important method to identify and quantify protein abundance. We performed LC-MS/MS to identify differentially expressed proteins using serum samples from healthy people and IBD patients. Among the identified proteins, there were 10 significantly altered proteins, 8 of which were related to immune function, suggesting that serum exosomes played an essential role in immune regulation in IBD. Many studies have also reported that exosomes are important mediators of the immune response [33]. In patients with intestinal inflammation such as IBD, the location and severity of the inflammation can change over time. In the pathogenesis of IBD, different types of cells (including intestinal epithelial cells and immune cells) may be activated [34] and communicate by releasing exosomes into the extracellular space. These exosomes can then circulate in the blood and be absorbed by macrophages in the gut, mediating their activation and triggering inflammation [10].

PZP is a high molecular weight glycoprotein that was initially described as elevated in the serum of women during pregnancy. PZP synthesis is estrogen-dependent, and it can be detected in serum a few weeks after incubation, reportedly returning to almost undetectable levels immediately after delivery. The immunosuppressive effects of PZP were profound. A study showed that intravenous infusion of PZP was sufficient to prevent heart transplant rejection in mice. Nevertheless, PZP knockout mice showed increased susceptibility to viral infection [35]. In addition, increased PZP concentration in the sputum is associated with respiratory tract infection caused by *Pseudomonas aeruginosa* [23]. In hepatocellular carcinoma, the expression of PZP was low in tumor tissue, and the down-regulation of PZP is associated with a poor clinical prognosis [36]. Recent studies found that PZP mRNA was downregulated in lung cancer tissues and was significantly correlated with immune cell infiltration. PZP as a new serum biomarker could identify lung adenocarcinoma in type 2 diabetes mellitus patients [37]. When proteomics was used to study the protein composition of plasma exosomes in healthy people, it was found that PZP protein was ubiquitous in exosomes [38], by which it could be delivered from donor cells to recipient cells, and regulated the function of recipient cells [8]. Reports showed that the release of exosomes from colon cancer cells stimulates the differentiation of adjacent fibroblasts into cancer-associated fibroblasts and myofibroblasts, thereby promoting angiogenesis and metastasis of colon cancer cells [39]. In this study, using LC-MS/MS, we found that the serum exosomes of IBD patients carried a high PZP level, which was 7 times higher than that in the healthy group, and we conducted further validation by Western blot. Considering that PZP might be a new serological diagnostic indicator of IBD, immunological detection was the preferred method for clinical verification and clinical practice [40]. Therefore, we used ELISA to detect the level of IBD in serum exosomes. The results of the three methods are consistent, showing that PZP protein levels increased significantly in serum exosomes of IBD patients.

The cargos carried by circulating exosomes of patients with various diseases may change due to the production of diseased cells [41]. Circulating exosomes mainly come from cell secretion. Hence, we predicted that the colon cells of IBD might secrete exosomes, and the colon-derived exosomes might be one of the factors affecting serum exosomes. Therefore, we used the acute colitis mouse model to investigate the expression of PZP in colonic exosomes and serum exosomes. Our animal experiment showed that the content of PZP protein in colonic exosomes of IBD was significantly increased, which was consistent with the results of serum exosomes. On the other hand, IBD is characterized by uncontrolled activation of intestinal immune cells in a genetically susceptible host [42], and PZP was highly expressed in immune cells [37]. Our experiments partly confirmed our conjecture that there might be a correlation between exosomes in the colon and serum. Based on literature reports and our experimental data, we hypothesized that in IBD patients, colon cells might release exosomes with abundant PZP into the bloodstream. However, the specific source of PZP needs further study.

## Conclusions

In summary, we found that the level of serum exosome PZP was much higher in patients with IBD. It might play a role in mediating immunity and inflammation in IBD patients through the protection and long-distance transport of exosomes. The mechanism of PZP in the occurrence and development of IBD remains to be further explored. Due to its high level in serum exosomes, PZP could be regarded as a promising indicator of serological detection for IBD diagnosis.

## Data Availability

Not applicable.

## Abbreviations

IBD: Inflammatory bowel disease
LC-MS/MS: Label-free liquid chromatography/mass spectrometry
PZP: Pregnancy zone protein
HCD: High energy collision dissociation
AGC: Automatic gain control
LFQ: Label-free quantification
FDR: False discovery rate
HC: Healthy controls
NAT: Nanoparticle Tracking Analysis
GO: Gene Ontology
DSS: Dextran sulfate sodium
